# MTML-msBayes: Approximate Bayesian comparative phylogeographic inference from multiple taxa and multiple loci with rate heterogeneity

**DOI:** 10.1186/1471-2105-12-1

**Published:** 2011-01-03

**Authors:** Wen Huang, Naoki Takebayashi, Yan Qi, Michael J Hickerson

**Affiliations:** 1 Biology Department, City University of New York, Queens College, 65-30 Kissena Blvd, Flushing, NY 11367-1597, USA; 2 Institute of Arctic Biology and Department of Biology and Wildlife, 311 Irving I Bldg, University of Alaska, Fairbanks, AK 99775, USA; 3 The Graduate Center of the City University of New York, 365 5th Ave, New York, NY 10016, USA

## Abstract

**Background:**

MTML-msBayes uses hierarchical approximate Bayesian computation (HABC) under a coalescent model to infer temporal patterns of divergence and gene flow across codistributed taxon-pairs. Under a model of multiple codistributed taxa that diverge into taxon-pairs with subsequent gene flow or isolation, one can estimate hyper-parameters that quantify the mean and variability in divergence times or test models of migration and isolation. The software uses multi-locus DNA sequence data collected from multiple taxon-pairs and allows variation across taxa in demographic parameters as well as heterogeneity in DNA mutation rates across loci. The method also allows a flexible sampling scheme: different numbers of loci of varying length can be sampled from different taxon-pairs.

**Results:**

Simulation tests reveal increasing power with increasing numbers of loci when attempting to distinguish temporal congruence from incongruence in divergence times across taxon-pairs. These results are robust to DNA mutation rate heterogeneity. Estimating mean divergence times and testing simultaneous divergence was less accurate with migration, but improved if one specified the correct migration model. Simulation validation tests demonstrated that one can detect the correct migration or isolation model with high probability, and that this HABC model testing procedure was greatly improved by incorporating a summary statistic originally developed for this task (Wakeley's *Ψ_W_*). The method is applied to an empirical data set of three Australian avian taxon-pairs and a result of simultaneous divergence with some subsequent gene flow is inferred.

**Conclusions:**

To retain flexibility and compatibility with existing bioinformatics tools, MTML-msBayes is a pipeline software package consisting of Perl, C and R programs that are executed via the command line. Source code and binaries are available for download at http://msbayes.sourceforge.net/ under an open source license (GNU Public License).

## Background

Comparative phylogeographic inference of multiple codistributed taxa is of central importance in evolutionary biology, biogeography and community ecology [1-5]. Soon it will be routine to use large amounts of genetic data sampled from multiple individuals and multiple non-model taxa [6] in combination with other sources of environmental and ecological information to make powerful biogeographic inference such as how climate change affects whole biota or how geographic processes generate and partition patterns of biodiversity across communities [7]. However, simultaneous analysis of data from multiple taxa and multiple unlinked loci presents analytical and computational challenges. By utilizing simulation and summary statistics to avoid the need to calculate an explicit likelihood function, ABC (approximate Bayesian computation) or "likelihood-free" methods can potentially tackle complex multi-taxa demographic models when more exact methods are not efficient [8]. Although some information in the data is sacrificed when only using summary statistics, ABC methods have been shown to compare well against methods that utilize an explicit likelihood function [9-11] by allowing efficient extraction of information from the data under explicit models that can be built from background information [12-14].

Here we present MTML-msBayes, a computer software pipeline that can be used to test for simultaneous divergence and migration across multiple codistributed taxon-pairs given multi-locus DNA sequence data. This method uses a coalescent-based model involving multiple taxa that can diverge at different times into taxon-pairs and independently experience different magnitudes of migration, population size-changes, and intra-genic recombination. The hierarchical model allows for variation and uncertainty in demographic parameters across taxon-pairs while testing specified multiple taxa scenarios of post-divergence migration and estimating hyper-parameters that characterize the variability in divergence times across taxon-pairs. Uncertainty in mutation rate heterogeneity across loci is also accounted for. For example, this software will allow testing for simultaneous divergence [11] and choosing among alternate multi-taxon scenarios such as isolation and migration that can be generated via ecological niche models [15]. Some recent packages have recently made ABC methods accessible to empiricists conducting phylogeographic inference [16-21], and MTML-msBayes complements these by using HABC for comparative phylogeographic datasets.

### Hierarchical Bayesian models

The use of a hierarchical Bayesian framework within the context of ABC has been described elsewhere [10-12,22,23]. As in the single locus msBayes [24], our hierarchical Bayesian model includes taxon-specific demographic parameters and locus-specific mutation parameters (*Φ*) that are conditional on demographic and mutational "hyper-parameters" (*ϕ*) which quantify the variability of *Φ *among the different taxon-pairs and loci. These hyper-parameters *ϕ*, can in turn be conditional on discrete "model indicator parameters" (*ϕ^Z^*). For example, taxon-specific parameters (*Φ*) for migration rates can vary across a set of population pairs conditional on either hyper-prior distributions *ϕ*^*1 *^or *ϕ*^*2*^, which both represent different biogeographic hypotheses about the dynamics of isolation across codistributed taxon-pairs. Potentially, Bayesian model choice can first be performed by obtaining the Bayesian posterior probabilities of models *ϕ*^*1*^, ..., *ϕ^Zmax ^*and subsequently obtaining the posterior probabilities of other hyper-parameters conditional on the model with highest posterior probability or averaged across models conditional on their relative posterior probabilities [22,25].

### Hierarchical ABC

Instead of explicitly calculating the likelihood expression P(Data|*ϕ^Z^*, *Φ*) to get a joint posterior distribution, we sample from the joint posterior distribution P((*ϕ^Z^*, *Φ*)|Data) by simulating the data under a coalescent model using candidate parameters drawn from the joint prior distribution P(*ϕ^Z^*, *Φ*). A summary statistic vector **D **of each simulated multi-taxon multi-locus dataset is then compared to the observed summary statistic vector D* in order to generate random observations from the joint posterior. MTML-msBayes implements hierarchical ABC by way of a standard rejection/acceptance algorithm [10,26-30] followed by an optional transformation step.

Specifically, for the simulated *i*th data set, a set of parameter values and *Φ_i _*are randomly drawn from their joint prior P(*ϕ^Z^*, *Φ*) and are then used to simulate data and associated **D**_*i*_. This is repeated until a large number of sample points from the joint prior distribution P(**D**,*ϕ^Z^*, *Φ*) have been obtained (typically 10^6 ^- 10^7^). The joint posterior distribution for *ϕ^Z ^*and *Φ *is sampled with probabilities proportional to the similarity between D* and each simulated sample of **D**_*i*_. The summary statistics within each vector **D**_*i *_are scaled to have unit variance followed by calculating a Euclidian distance between **D**_*i *_and D*. Subsequently, a user-defined proportion of simulated summary statistic vectors **D**_*i *_are accepted with their associated parameter values being used to sample the joint posterior. Typically 500-10,000 simulated data sets are accepted out of > 10^6 ^prior simulations. To improve upon the posterior estimation, an optional transformation step can involve local linear regression for continuous hyper-parameters following the scheme of [31] or polychotomous logistical regression for estimating discrete model indicator parameters or discrete integer hyper-parameters [25,32,33]. Alternatively, one could apply other post-acceptance transformation methods [21,34,35] such as the general linear model [21].

### Models of demography and DNA sequence evolution

**Figure 1:**
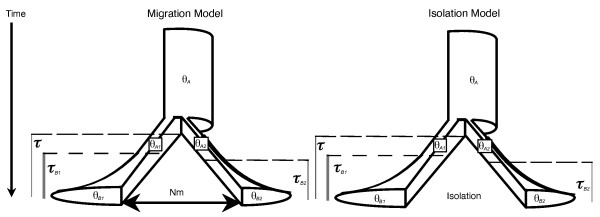
**Depiction of isolation and migration models of a taxon diverging into sister taxa**. Up to *Y *taxon-pairs diverge at 1 to Ψ different divergence times where all parameters shown are free to vary across the *Y *taxon-pairs. Additional file 1 summarizes all the parameters in the multi-taxon-pair model of divergence used in MTML-msBayes.

MTML-msBayes generates finite sites DNA sequence data simulated under a coalescent demographic model to perform ABC. The data generation step is accomplished by msDQH which is a version of Hudson's classic coalescent simulator which simulates finite sites DNA sequence data under specified demographic model [36]. The general multiple taxon-pair hierarchical ABC model of divergence with migration and size change that can be implemented in MTML-msBayes is presented elsewhere [11,24] and generally involves using the multiple taxon-pair data to estimate hyper-parameters and parameter summaries that quantify the variability in divergence times across *Y *taxon-pairs (Additional File 1; Figure 1). This includes Ψ, the number of different divergence times across *Y *taxon-pairs, which follows a discrete uniform prior distribution ranging from 1 to *Y*. Additionally one can estimate the mean divergence time, E(*τ*), where *τ *is the time of divergence between a pair of descendent population pairs (in coalescent time units of 4*N *generations, where *N *is the sum of current effective population sizes of the two descendent sister populations), as well as estimate Ω, the dispersion index of *τ*, (Var(*τ*)/E(*τ*)). If one conducts the analysis using a partially constrained model where the number of divergence times (Ψ) is held to a single value across the *Y *taxon-pairs, one can subsequently estimate each of the Ψ divergence times (*τ_1_*, ..., *τ_Ψ_*), as well as the number of taxa that split at each of the Ψ times (Ψ_*1*_, ...,Ψ_*Ψ*_). Following the algorithm detailed in [11,24], the Ψ divergence times *τ_1_*, ..., *τ_Ψ _*are randomly drawn from a user-specified uniform prior distribution and these Ψ divergence times are subsequently assigned randomly to Ψ taxon-pairs of the *Y *taxon-pairs with the remaining *Y *- Ψ taxon-pairs randomly picking divergence times from *τ_1_*, ..., *τ_Ψ _*with replacement.

As in [24], each taxon consists of an ancestral population of effective size *θ_A _*that splits at time *τ *into two descendent populations of effective sizes *θ_A1 _*and *θ_A2 _*which then start exponentially growing into sizes *θ_B1 _*and *θ_B2 _*at times *τ_B1 _*and *τ_B2_*. If there is migration incorporated into the demographic model, each taxon-pair has an effective migration rate that occurs after divergence (*Nm*; where *m *is the probability of symmetric migration between pairs of descendent sister populations). The parameters *Nm, θ_A_*, *θ_A1_*, *θ_A2_*, *θ_B1_*, *θ_B2_*, *τ_B1 _*and *τ_B2 _*all independently vary across all codistributed taxon-pairs according to uniform prior distributions that are specified by the researcher.

The multiple loci from each taxon-pair are assumed to be unlinked and for the mutation model, the Jukes-Cantor [37], equal-input (F81) [38], or HKY model [39] of DNA substitution can be optionally used for all loci [37], with the equal-input model being default. Because the divergence with migration model is generally applied to taxa that have split in the last 10 My, these models should be sufficient [40]. The rate of DNA substitution can vary across unlinked loci such that the rate differences are scaled from the mean of a gamma distribution. Uncertainty in the shape parameter α, is incorporated by randomly drawing α from a uniform hyper-prior distribution ranging between 1 and 20 with the scale parameter = 1/α, such that the mean rate scalar is 1.0 across replicate simulations. If there is prior evidence regarding specific patterns in rate variation amongst loci, such as relative rate estimates from samples of outgroup taxa, one can constrain loci to have specific average rate differences. Furthermore, a scalar parameter for each locus can incorporate ploidy differences for loci such as haploid uniparentally inherited mitochondrial and chloroplast DNA, and likewise these scalar parameters can enforce relative differences in generation times across taxon-pairs. A uniform prior distribution can be optionally specified for intragenic recombination rates that vary independently across taxa.

### Summary Statistics

The summary statistic vector **D **in MTML-msBayes can calculate up to 23 summary statistic classes collected from each locus of every taxon-pair. These summary statistic classes are of three categories: 1.) whole population summary statistics that treat the taxon-pair as a single population sample; 2.) subpopulation summary statistics that are calculated on each of the two descendent population samples, and 3.) summary statistics that quantify differences between the two descendent population samples. Categories 1 and 2 include *π*, the average number of pairwise differences among all sequences within each population pair, *θ_W _*the number of segregating sites within each population pair normalized for sample size, [41], SD(*π *- *θ_W_*) the standard deviation in the difference between these two quantities, *sH*, Shannon's diversity index on allele frequencies [42], and *s*, Wakeley's population correlation coefficient in the number of pairwise differences [43]. Category 3 includes, *π_b _*and *π_net_*, the total average and net average pairwise differences between two descendent population samples, [44], and Wakeley's *s_XY _*and *Ψ_W_*, two derivations of the interpopulation correlation coefficient in the number of pairwise differences between populations. These latter two summary statistics have been demonstrated as useful for distinguishing migration from isolation models [43,45].

For every simulated dataset of multiple taxon-pairs and multiple loci, a three dimensional vector (**D**) of these summary statistics is first constructed with dimensions of ***x ***summary statistic classes, ***y ***taxon-pair indicator elements and ***z ***loci. Subsequently, a new 3-dimensional vector **D_*m *_**is calculated from **D **where ***z_m _***consists of the first four raw moments across loci [46]. The raw moments are the moments about zero, which can be converted to central moments (the first to forth central moments correspond to mean, variance, skewness, and kurtosis, respectively) using binomial transformation [47]. Moments are used to reduce the dimensionality of summary statistics vector and to capture the distribution of random variables (summary statistics) across loci. To be general, the number of sampled loci can vary amongst taxon-pairs such that the length of ***z ***varies within **D **whereas within **D_*m*_**, ***z ***can have up to 4 elements.

When calculating **D_*m*_**, a final step involves re-ordering the taxon-pair indicator elements of ***y ***into descending values of mean *π_b _*across loci such that each of the **x **columns of summary statistic classes have their **y **elements tandemly ordered by descending values of *π_b_*. This greatly reduces the combinatorial sample space such that order of the original sampling configuration is not determinant on any corresponding ordered vector of *π_b_*'s (which are predicted to correlate with the corresponding vector of *τ*'s [48]). This strategy takes advantage of the *exchangeability *of the expected values of *π_b _*across sample sizes and their correlation with each taxon-pair's *τ *(divergence time) [48]. By using this re-ordering procedure, the Euclidian distance between each simulated (**D_*m*_**)_*i *_and observed D_m_* is independent of the ordering of taxon-pairs within the sampling configuration and results in a higher correlation between ΔΩ and Δ**D_*m *_**than when not re-ordering. Here, ΔΩ is the difference in Ω (dispersion index of divergence times across *Y *taxon pairs) between pairs of data sets and Δ**D_*m *_**is the Euclidian distance between their corresponding pairs of summary statistic vectors **D_*m *_**that are calculated from these corresponding pairs of data sets. This ordering scheme for **D_*m *_**results in a desired ABC strategy with a higher correlation between summary statistics and estimated parameters (i.e. Ω and **D_*m*_**). This was confirmed by comparing pairs of simulated data sets and here we verify the improved performance of this sorting procedure via simulations.

### Implementation

Running MTML-msBayes is a four step process that includes: (1) preparation of the input file specifying prior distributions and the sampling configuration from the DNA sequence data; (2) preparation of the observed summary statistic vector, (3) generating a "reference table" of simulated data sets (i.e. coalescent simulations of data sets matching the observed data with regards to the sampling configuration and with parameters drawn from the prior); and (4) performing an acceptance/rejection step to obtain a sample from the posterior distribution. To improve estimation, accepted parameter values sampled from the posterior distribution can be subjected to transformation methods depending on if whether they are continuous parameters (local linear regression) or discrete parameters (polychotomous regression) using R scripts provided by M. Beaumont. Alternatively, one could perform other recently developed methods of post-acceptance transformation to improve parameter estimation [21,34,35].

Due to the modular pipeline architecture of MTML-msBayes, users can also opt to use available command line driven R scripts to generate pseudo-observed data sets (i.e. "PODS"; [14]) in order to conduct simulation-based model validation as well as fine tune the ABC conditions with respect to choice of summary statistics and acceptance threshold. In addition, users can use available R scripts to conduct posterior model fitting in order to assess the fit of the simulation models to the observed data [13,14].

After installing the software via binary installation or compilation of source code, each of the four main steps is performed by executing four corresponding Perl executables on the command line. The data can be formatted as IM files [49], or FASTA files. While the data configuration file now accommodates multiple locus data, MTML-msBayes can analyze single locus data sets thereby superceding the previous single-locus msBayes. We distribute MTML-msBayes as C source code, R scripts and Perl executables to be run on the command line after compiling on Linux, Mac OS-X, and most POSIX systems using instructions from the README file. The MTML-msBayes package is available from http://msbayes.sourceforge.net/ and also includes an online manual with installation/running instructions available from https://docs.google.com/Doc?docid=0AVkCIu87W8ooZGNyc3M2ZDhfNDJkZm5zd3dmcg&hl=en.

## Results

**Figure 2:**
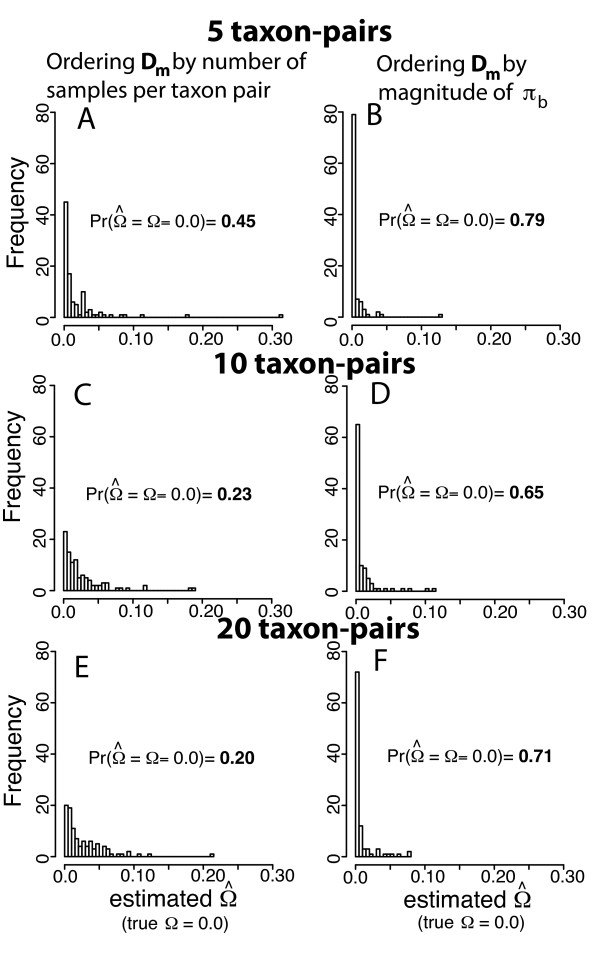
**Comparison of sorting algorithms for summary statistic vector D_m_**. Frequency histograms depicting sets of 100 ABC estimates of Ω given PODS simulated under simultaneous divergence (Ω = 0; Ψ = 1) using two different algorithms for ordering the taxon-pair elements of ***y ***within **D_m _**(panels A, C, and E by number of samples per taxon-pair; panels B, D, and F by the magnitude of the mean value of *π_b _*across loci). Results are presented for data sets that correspond to 5, 10 and 20 taxon-pairs. Each point estimate is the mode of 500 accepted points in total out of 1,500,000 simulated data sets using ABC with local linear regression.

**Figure 3:**
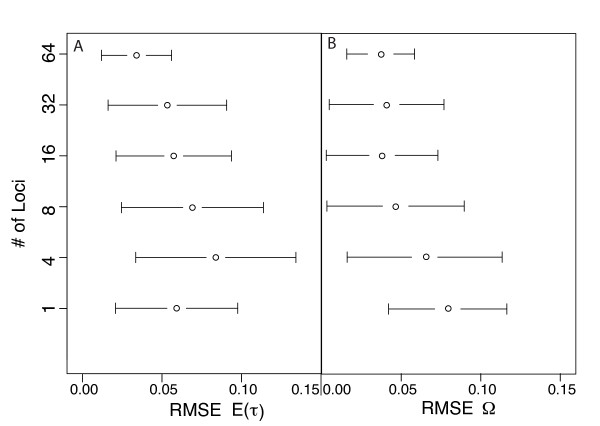
**RMSE: ABC algorithm validation for estimator bias and precision**. RMSE (root mean square error) across 100 estimates of parameter values given 100 PODS (pseudo observed data sets) simulated with known parameter values. Panel A corresponds to estimates of E(*τ*) and panel B corresponds to estimates of Ω. The error bars depict 2 × SD (standard deviation) of the RMSE across each set of 100 estimates. For all PODS, Ψ (number divergence times across five taxon-pairs) is drawn from its discrete uniform hyper-prior ranging between 1 (simultaneous divergence) and 5 (the number of taxon-pairs). PODS and corresponding priors were simulated given data from 1, 4, 8, 16, 32 and 64 loci each from 5 taxon-pairs. Each RMSE is calculated from the 100 true hyper-parameter values (E(*τ*) and Ω) and the corresponding 100 posterior mode estimates (mode from the 500 accepted points out of a total 1,500,000 draws from the hyper-prior using ABC with local linear regression and a summary statistic vector **D_m _**that only included mean values of *π_b _*across loci from every taxon-pair).

**Figure 4:**
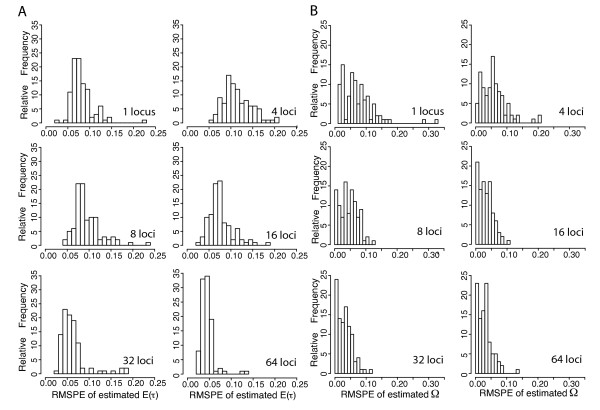
**RMSPE: ABC algorithm validation for estimator bias and precision**. Histograms depicting the distribution of RMSPE (root mean square posterior error) for 100 estimates of parameter values given 100 PODS (pseudo observed data sets) simulated with known parameter values. Panel A corresponds to estimates of E(*τ*) and panel B corresponds to estimates of Ω. For all PODS, Ψ (number divergence times across five taxon-pairs) is drawn from its discrete uniform hyper-prior ranging between 1 (simultaneous divergence) and 5 (the number of taxon-pairs). PODS and corresponding priors were simulated given data from 1, 4, 8, 16, 32 and 64 loci each from 5 taxon-pairs. Each RMSPE is calculated from the true hyper-parameter value (E(*τ*) and Ω) and the corresponding 500 accepted points out of a total 1,500,000 draws from the hyper-prior using ABC with local linear regression and a summary statistic vector **D_m _**that only included mean values of *π_b _*across loci from every taxon-pair.

**Figure 5:**
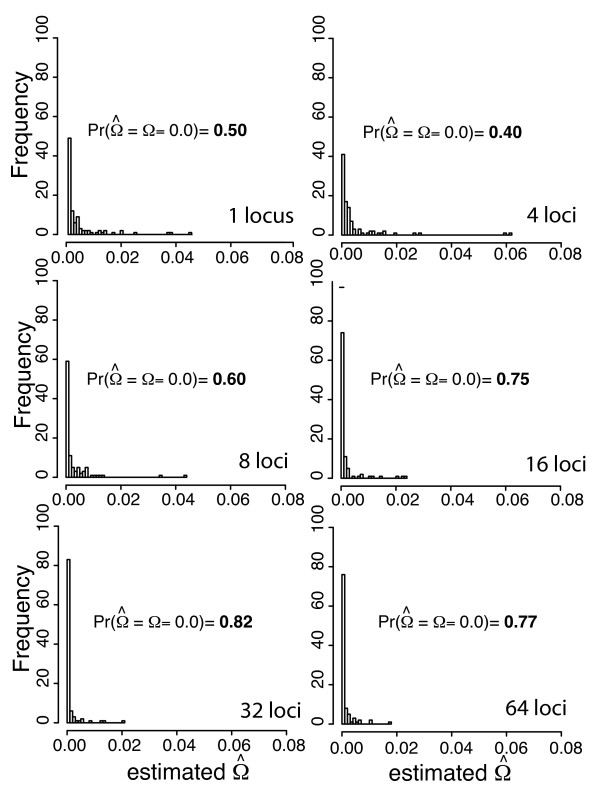
**ABC algorithm validation for estimator accuracy under simultaneous divergence**. Frequency histograms of sets of 100 ABC estimates of E(*τ*) (panel A) and Ω (panel B) with each test data set simulated under simultaneous divergence (Ω = 0; Ψ = 1) and each simulated draw from the hyper-prior had the number divergence times across five taxon-pairs (Ψ) drawn from its discrete uniform hyper-prior between 1 (simultaneous divergence) and 5 (the number of taxon-pairs). Test data and corresponding priors were simulated given data from 1, 4, 8, 16, 32 and 64 loci each from 5 taxon-pairs. Each point estimate is the mode of 500 accepted points in total out of 1,500,000 simulated data sets using ABC with local linear regression and a summary statistic vector **D_m _**that only included mean values of *π_b _*across loci from every taxon-pair.

**Figure 6:**
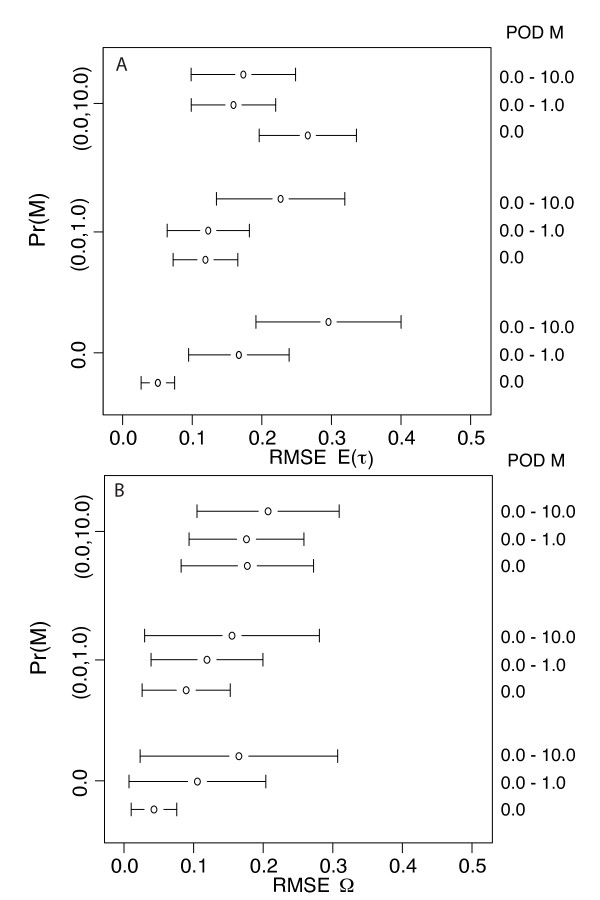
**RMSE: ABC algorithm validation given different levels of assumed and known migration rates and D_m _= *π_b_***. RMSE (root mean square error) across 100 estimates of parameter values given 100 PODS (pseudo observed data sets) simulated with known parameter values. Panel A corresponds to estimates of E(*τ*) and panel B corresponds to estimates of Ω. The error bars depict 2 × SD (standard deviation) of the RMSE across each set of 100 estimates. For all PODS, Ψ (number divergence times across five taxon-pairs) is drawn from its discrete uniform hyper-prior ranging between 1 (simultaneous divergence) and 5 (the number of taxon-pairs). PODS and corresponding priors were simulated given 16 loci each from 5 taxon-pairs. Three different hyper-priors were used with respect to post-divergence migration rates as well with simulating PODS (migration rate *Nm *= 0, 0-1, and 0-10 migrants per generation where migration rate varies independently across taxon-pairs within each 5 taxon-pair data set). Each RMSE is calculated from the 100 true hyper-parameter values (E(*τ*) and Ω) and the corresponding 100 posterior mode estimates (mode from the 500 accepted points out of a total 1,500,000 draws from the hyper-prior using ABC with local linear regression and a summary statistic vector **D_m _**that only included mean values of *π_b _*across loci from every taxon-pair).

**Figure 7:**
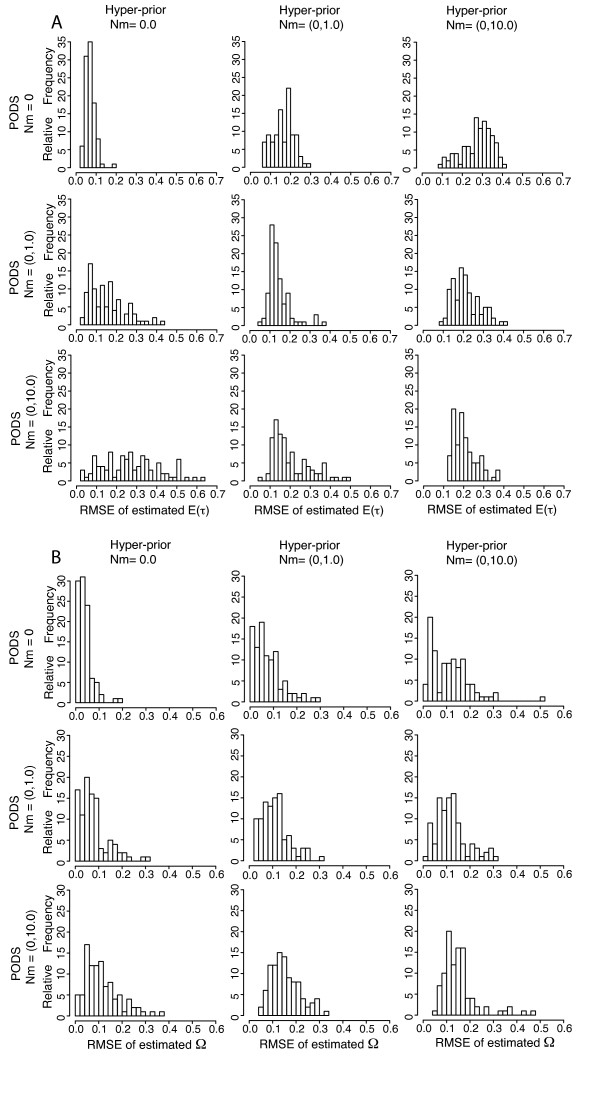
**RMSPE: ABC algorithm validation given different levels of assumed and known migration rates and D_m _= *π_b_***. Histograms depicting the distribution of RMSPE (root mean square posterior error) for 100 estimates of parameter values given 100 PODS (pseudo observed data sets) simulated with known parameter values. Panel A corresponds to estimates of E(*τ*) and panel B corresponds to estimates of Ω. For all PODS, Ψ (number divergence times across five taxon-pairs) is drawn from its discrete uniform hyper-prior ranging between 1 (simultaneous divergence) and 5 (the number of taxon-pairs). PODS and corresponding priors were simulated given 16 loci each from 5 taxon-pairs. Three different hyper-priors were used with respect to post-divergence migration rates as well with simulating PODS (migration rate *Nm *= 0, 0-1, and 0-10 migrants per generation where migration rate varies independently across taxon-pairs within each 5 taxon-pair data set). Each RMSPE is calculated from the true hyper-parameter value (E(*τ*) and Ω) and the corresponding 500 accepted points out of a total 1,500,000 draws from the hyper-prior using ABC with local linear regression and a summary statistic vector **D_m _**that only included mean values of *π_b _*across loci from every taxon-pair.

To ascertain how well MTML-msBayes quantifies the congruence of divergence times under a number of different conditions, we conduct an extensive simulation analysis by generating PODS (pseudo-observed data sets; [14]) and quantifying the accuracy and precision of estimates on the known parameter values used to generate the PODS. Specifically, we assessed: 1.) the advantage of re-ordering elements of ***y ***(taxon-pair indicators) within **D_m _**by descending magnitude of *π_b _*averaged across loci with respect to estimating Ω as a function of number of taxon-pairs (*Y*) within the sample (Figure 2); 2.) the effect of increasing numbers of loci (1, 4, 8, 16, 32, and 64 loci) when estimating E(*τ*) and Ω (Figures 3, 4, and 5); 3.) the consequences of allowing for and ignoring rate heterogeneity across loci (Additional file 2); and 4.) how different levels of post-divergence migration influence estimates of E(*τ*) and Ω and how this is influenced by migration/isolation model misspecification (Figures 6 and 7).

For simulation-based testing, we generally compare estimates from PODS with the known hyper-parameter values that simulated the PODS [10,50] and calculate RMSE and RMSPE (root mean square error and root mean square posterior error) using these known values and each posterior mode estimate and the of 500 accepted posterior hyper-parameter values in order to gauge the amount of uncertainty and bias associated with posterior estimates. PODS are simulated using random draws from the hyper-prior of Ψ, where Ψ ranges from 1 to *Y *according to a discrete uniform distribution or alternatively are simulated under a history of simultaneous divergence (Ψ = 1; Ω = 0.0). For each set of conditions (i.e. number of loci, migration levels or chosen **D_m_**) we conduct ABC on sets of 100 independently generated PODS and for each we estimate E(*τ*) and Ω. For each set of 100 PODS and set of conditions we recycle the same 1,500,000 random draws from the prior (reference table), and use 500 accepted draws for ABC posterior estimation. In all cases, the simulated prior and sets of 100 PODS matches exactly with respect to sample size (i.e. number of loci and taxon-pairs). Simulated data included of 5 to 20 taxon-pairs and 1 - 64 loci whose length was 1100 base-pairs.

Additionally we include an empirical analysis of three Australian avian taxon-pairs that are hypothesized to have arisen simultaneously from three codistributed ancestral species due to the emergence of the Carpentarian barrier in northern Australia [51,52]. Specifically, the three taxon-pairs consist of the red backed fairy wren, *Malurus melanocephalus melanocephalus *and *M. m. cruentatus *(37 loci of 58 - 467 base pairs and mean of 27.8 individuals per descendent sister taxon), the black-throated and long-tailed finches, *Poephila cincta *and *P. acuticauda *(30 loci of 216 - 650 base pairs and one individual collected per descendent sister taxon) and the brown and black-tailed treecreepers, *Climacteris picumnus *and *C. melanura *(15 loci of 201 - 358 base pairs and mean of 9.5 individuals per descendent sister taxon).

### Summary statistic vector D_m_

When looking at pairs of PODS, the values of Δ**D_*m *_**between the pairs of simulated summary statistic vectors will be correlated with ΔΩ under optimal conditions of estimating Ω. Likewise when Ω is fixed at 0.0 (simultaneous divergence), values of Δ**D_*m *_**should be 0.0 under such optimal conditions for estimating Ω. To verify that ordering elements of ***y ***(taxon-pair indicators) by the first moment of *π_b _*leads to more accurate estimates of Ω under simultaneous divergence than when ordering ***y ***(taxon-pairs) by arbitrary order defined in the sampling configuration, we conduct simulation tests and plot frequency histograms of estimates of Ω given that PODS are generated under simultaneous divergence (Figure 2). Not only is the strategy for re-ordering **D_*m *_**superior to ordering arbitrarily, this advantage becomes more substantial as the number of taxon-pairs increase (Figure 2D and 1F). Due to the *exchangeability *of *π_b _*across sample sizes, this sorting strategy minimizes Δ**D_*m *_**between observed and simulated data in cases when ΔΩ = 0.0 and Ω = 0.0 (simultaneous divergence). The increasing advantage of this re-ordering strategy as the number of sampled taxon-pairs increases is expected given that ordering by the magnitude of *π_b _*obviates the need to simulate from the entire combinatorial sample space with regards to all possible orders from which the taxon-pairs could be simulated when making the prior. Because this combinatorial sample space quickly increases with number of taxon-pairs, ordering by some arbitrary rule such as number of samples per taxon-pair results in increasing magnitudes Δ**D_*m *_**with greater number of taxon-pairs given that Ω = 0.0 (Figure 2). Although using only the mean of *π_b _*across loci results in reasonable estimates of Ω, other available summary statistics are available for future applications of MTML-msBayes that will involve testing complex multi-species histories other than simultaneous divergence. When this software pipeline is expanded to allow data consisting of large numbers of SNPs such that none of the information in the data are lost, we expect that a strategy involving genetic distances instead of Euclidian distances might work well for testing multi-taxa hypotheses or alternatively using the first four moments of sets of summary statistics calculated across loci and/or taxa [46,53]. For further information about the bourgeoning set of methods and strategies being developed for ABC, [12-14] provide thorough overviews.

### Number of loci

As expected, increasing numbers of loci lead to more accurate estimates of Ω (Figures 3B, 4B and 5). However, improvement in estimation of E(*τ*) is not a monotonic increase with the number of loci (Figures 3A and 4A). The performance of estimating E(*τ*) with 4 loci is worse than a single locus, and the advantage of more loci is not reached until ≥16 loci are used (Figures 3A and 4A). In this case, sufficient sampling of loci is required to overcome the variance introduced by rate heterogeneity across loci. Estimating Ω on the other hand improves with 8 loci and continues to improve. We note that the accuracy of both estimators improves substantially with > 32 loci (Figures 3, 4, and 5).

### Rate heterogeneity

Overall, estimating both Ω and E(*τ*) was relatively insensitive to whether or not the model of across-locus rate heterogeneity was correctly specified (Additional file 2). Generally, estimator performance was highest when the PODS had equal rates, but we note that PODS with unequal rates resulted in high accuracy in estimates of Ω and E(*τ*) irregardless of whether rate heterogeneity or rate uniformity was correctly specified in the prior model.

### Post-divergence Migration

Given data sets with 16 loci per each of five taxon-pairs, migration had a negative impact on the estimation of Ω and E(*τ*) but the magnitude of this negative impact depended on whether one used the correct migration model for simulating the prior. As theory predicts [54-56], we generally found that estimates of Ω and E(*τ*) became less reliable with increasing migration (Figures 6 and 7) even when the correct migration models were used. Migration model misspecification also influenced estimator bias and precision. When the PODS are generated under isolation, the estimators of Ω and E(*τ*) generally became less accurate with increasing migration model misspecification. Likewise, when PODS were generated under a migration model, model misspecification resulted in higher estimator bias and less precision as quantified by RMSE and RMSPE.

Overall, this simulation analysis demonstrates that quantifying the level of temporal congruence in multi-taxa divergence will be augmented if one first tests for migration so that an appropriate hyper-prior model can be specified. Therefore it would be wise to test whether a migration or isolation model is justified in the taxon-pairs using informational theoretic and MCMC techniques [56,57] or ABC model choice before quantifying the level of congruence in multi-taxa divergence. Overall, this simulation analysis demonstrates that our multi-locus test for simultaneous divergence will work better if one is interested in testing simultaneous cessation of all gene flow rather then testing for simultaneous divergence with some post-isolation gene flow. However, it remains to be seen whether larger numbers of loci and/or other summary statistics can better infer multi-taxa divergence with limited migration or secondary contact.

### Hierarchical ABC model choice among competing migration models

Because the accuracy of estimation can depend on assumptions about migration after divergence, one can first use ABC model choice techniques [32,58] to compare the posterior probability of isolation and post-divergence migration models in the context of our hierarchical multi-taxa divergence model. Specifically we treat models of isolation and migration as a set of models specified by a categorical model indicator parameter that can be estimated via ABC. In this case the acceptance rejection step can be followed by a polychotomous regression step that has been shown to improve estimation of discrete categorical parameters [15,22,25,32]. To test the accuracy of this technique, the five taxon-pair data was simulated using 3,000,000 random draws from the hyper-prior with the three different migration models simulating the data with equal probability (one isolation model and two migration models). For the two migration models, each of the five taxon-pair's migration parameter (*Nm*; number of effective migrants per generation) is independently drawn from a uniform prior distribution (0.0,1.0) or (0.0,10.0) depending on which of the two migration models. Subsequently the posterior for the model indicator parameter conferring to isolation or the two different migration levels (*Nm *upper bounds of 1.0 and 10.0) was sampled from the 500 closest accepted matches obtained with the ABC algorithm with and without subsequent polychotomous regression.

The accuracy of this ABC model choice procedure was then assessed by conducting this procedure on 100 PODS of five taxon-pairs and 16 loci simulated under each of the three different migration models (isolation and *Nm *upper bounds of 1.0 and 10.0). The probability of choosing the correct migration model ranged from 0.77 to 0.96 when one used a summary statistic vector **D_m _**that included the across loci mean π_b _and *Ψ_W _*(Additional file 3) whereas using π_b _only resulted in fewer successful model choices (probability of choosing correct model ranging from 0.57-0.72). Indeed, Wakeley's *Ψ_W _*was developed as a way to distinguish between migration and isolation models [43] and when harnessed within our hierarchical ABC framework, we demonstrate it to have potential application given a multiple taxon-pair model. Additionally, the use of polychotomous regression greatly improved the probability of successful model choice over using direct non-transformed accepted values. Likewise, the Bayes factor support for the correct model was augmented when using π_b _and *Ψ_W _*as well as polychotomous regression (Additional file 3).

### Empirical analysis

To demonstrate how MTML-msBayes can test for simultaneous divergence given large numbers of loci and post-divergence migration, we used 15-37 loci collected from three bird taxon-pairs all of which consist of sister taxon-pairs that presently span the Carpentarian barrier in northern Australia [51,52]. This includes the brown and black-tailed treecreepers (*Climacteris picumnus *and *C. melanura*), the black-throated and long-tailed finches (*Poephila cincta *and *P. acuticauda*) and the eastern and western ssp. of red-backed wrens (*Malurus melanocephalus melanocephalus *and *M. m. cruentatus*). Results strongly suggest that all three sister taxon-pairs diverged at the same time and hence could have formed by way of the same geo-climatic event that formed the Carpentarian barrier in northern Australia. Furthermore, this result of simultaneous divergence was insensitive to whether or not one incorporated low levels of migration after divergence. The time of this simultaneous divergence was 81,000 y.b.p. and 200,000 y.b.p. under isolation and low migration models respectively.

As expected from theory and shown in our simulation analysis (Figures 6 and 7), tests of simultaneous divergence and divergence time estimation are dependent on model assumptions about post-divergence migration [54-56] and therefore we initially used ABC model choice [32,58] to compare the posterior probability of two models; complete isolation and post-divergence migration across all three taxon-pairs (Figure 1). To generate simulated data for ABC, the three taxon-pair data set was simulated 3,000,000 times using random draws from the hyper-prior and both isolation and migration models were used to simulate the data with equal probability. Under the migration model, the three values of *Nm *for each of the three taxon-pairs (number of effective migrants per generation) are independently drawn from a uniform prior distribution (0.0,1.0) and assigned to each taxon-pair. After conducting the ABC model selection, the low migration model had more support (Pr(migration | ***D_m_***) = 0.65)), yet not enough for "high" or "moderate" Bayes factor support [59]. Alternatively, we estimated E(*τ*) and Ω from mixed isolation/migration priors such that estimates of E(*τ*) and Ω are averaged across the relative posterior probability of the isolation model and migration model. In this case, the goal is not to test the models but to obtained estimates of E(*τ*) and Ω while allowing uncertainty in model selection.

**Figure 8:**
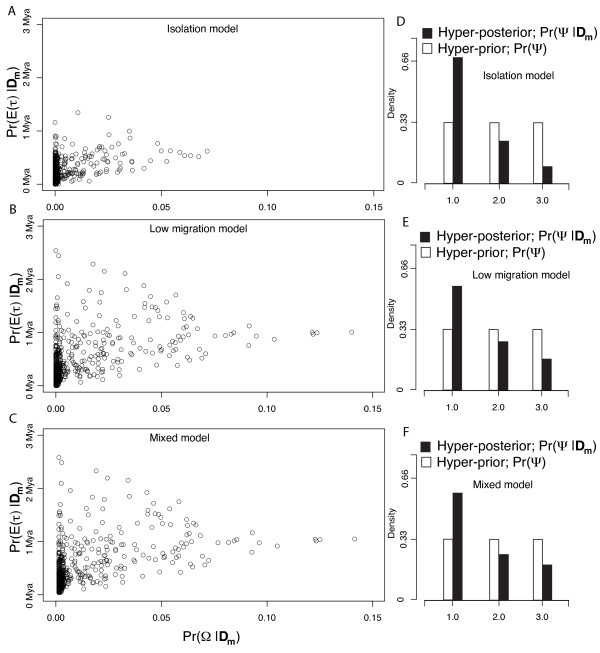
**Estimates of the mean, dispersion index and number of divergence times given empirical data**. Panels A, B and C depict joint posterior densities of two hyper-parameter summaries that characterize the average divergence time (E(*τ*)) and dispersion index of divergence times Ω = Var(*τ*)/E(*τ*)) across three avian taxon-pairs that span the Carpentarian barrier in northern Australia. Each point is from a data set simulated using parameters randomly drawn from the prior and subsequently accepted using ABC with local linear regression (500 accepted points in total out of 3,000,000 simulated data sets) and a summary statistic vector **D_m _**that only included mean values of *π_b _*across loci from every taxon-pair. Panels D, E, and F depict hyper-prior and hyper-posterior densities of Ψ, the number of divergence times across taxon-pairs. Panels A and D results are under a model of total isolation after divergence, panels B and E results are under a model allowing for low migration after divergence, with each taxon independently having *Nm = *0.0 - 1.0 between sister taxa after divergence. Panels C and F are results using a mixed model where the posterior is averaged across the two models while weighting for the relative posterior probability under the two models. Divergence times assume an average rate across loci of 5.0 × 10^-9 ^per site per generation and two year generation times.

Hyper-parameter estimates of Ψ and Ω indicate an inference of simultaneous divergence, with Ψ = 1 having the highest probability irregardless of which migration/isolation model is used, (Pr(Ψ = 1| ***D_m_***, isolation) = 0.67; Pr(Ψ = 1| ***D_m_***, migration) = 0.58; and Pr(Ψ = 1|***D_m_***, mixed model) = 0.60). Likewise, Ω (the dispersion index characterizing the variability in divergence times) indicated synchronous divergence irregardless of migration model with mode estimates of Ω = Var(*τ*)/E(*τ*)) = 0.0 across all three migration/isolation models (Figure 8). The resulting Bayes factor comparing models of simultaneous divergence (Ψ = 1) and non-simultaneous divergence (Ψ>1) did depend on whether migration was assumed with moderate support for simultaneous divergence given isolation (*B*(Ψ = 1,Ψ>1) = 4.05), weak support for simultaneous divergence given migration (*B*(Ψ = 1,Ψ>1) = 2.76) and the mixed model (*B*(Ψ = 1,Ψ>1) = 2.92). Consistent with our expectations that allowing migration will result in divergence time estimates with more uncertainty, the posterior estimates of mean divergence time and tests of simultaneous divergence are less precise under the low migration model than under a pure isolation model, and the posterior estimates of mean divergence time, (E(*τ*)), are older under migration than under isolation (Figure 8).

As always, translating scaled divergence time estimates into real time estimates depends on assumptions about DNA mutation rates and here we report real time estimates based on DNA mutation rates reported previously. An assumed mean rate across loci of 5.0 × 10^-9 ^per site per generation (as reported by [52]) and a two year generation time results in mean divergence time, E(*τ*) estimates of 81,000 y.b.p. and 200,000 y.b.p. under isolation and low migration models respectively. These estimates are generally consistent with the reported divergence time estimates of the wrens (Lee and Edwards 2008; 270,000 y.b.p) and finches (Jennings and Edwards 2005; 432,000 y.b.p.) using the same rates and a similar coalescent-based isolation with migration model that used Markov chain Monte Carlo [49,60]. We additionally note that Lee and Edwards [61] estimated low levels of migration (< 1.0 migrants per generation) in the fairy wrens which is also consistent with the higher posterior probability for the low migration model that we found via ABC model selection. Further, the older and less precise estimate of means divergence time under migration than under isolation is expected due to migration breaking up the correlation between coalescent times and divergence times [54-56].

## Conclusions

Multi-species comparative phylogeographic inference using genetic data from large numbers of non-model taxa will increasingly become a standard tool for understanding the interplay between geography, climate change, speciation, extinction, demographic changes, and species interactions as well as making links between different types of biodiversity, ecological services and ultimately well-informed conservation policy [62,63]. Inferring how whole assemblages of species react to putative geographical barriers is central to obtaining these larger goals and MTML-msBayes will become an important bioinformatics tool for such inference given multi-level models with large amounts of complexity. Phylogeographic data sets with multiple codistributed taxon-pairs with genetic data collected from multiple loci are rapidly emerging [64-67], and here we demonstrate that correct inference of simultaneous divergence is somewhat robust against violations in assumptions about among locus rate heterogeneity although incomplete isolation with post-divergence migration can make inference of simultaneous divergence difficult. Furthermore, it is likely that other demographic complexities such as pre-isolation subdivision, diminishing/accelerating levels of post-isolation migration, and recombination are likely to affect inference [68]. Although MTML-msBayes does optionally allow for intra-genic recombination, testing how ignoring this parameter biases inference is beyond the scope of this work and researchers should test for recombination or use non-recombinant blocks for analysis.

The modular design of MTML-msBayes further allows simulation-based model validation and posterior predictive model fitting and will be able to interface with other bioinformatics tools developed for ABC [20,21]. Moreover, the modular design will ultimately allow implementing various constrained analyses for testing an array of multi-taxon histories beyond the tests of migration and simultaneous divergence presented here so that researchers will finally be able to make large scale biogeographic inference across whole communities with sufficient demographic complexity.

## Availability and requirements

We distribute MTML-msBayes as C source code, R scripts and Perl executables under open-source, GNU General Public License to be run on the command line after compiling on Linux, Mac OS-X, and most POSIX systems using instructions from the README file. The MTML-msBayes package is available from sourceforge http://msbayes.sourceforge.net/ and also includes an online manual with installation/running instructions available from as well as associated R scripts to conduct simulation testing are available from http://qcpages.qc.cuny.edu/Biology/Hickerlab/Software/Software.html

## List of abbreviations

The abbreviations include ABC: (Approximate Bayesian Computation); HABC: (Hierarchical Approximate Bayesian computation); RMSE: (Root Mean Square Error); RMSPE: (Root Mean Square Posterior Error); and PODS: (Pseudo-Observed Data Sets).

## Authors' contributions

WH, NT, YQ, and MJH developed C, R, and Perl routines for the multi-taxa/multi-loci model with rate heterogeneity for ABC estimation and model choice. WH and MJH did the extensive simulation testing and MJH conducted the empirical analysis. MJH and NT maintain MTML-msBayes and NT developed the installation configurations. All authors read and approved the final version of the manuscript.
